# TREATMENT OF PERIOCULAR HYPERPIGMENTATION DUE TO LEAD OF KOHL (SURMA) BY PENICILLAMINE: A SINGLE GROUP NON-RANDOMIZED CLINICAL TRIAL

**DOI:** 10.4103/0019-5154.57614

**Published:** 2009

**Authors:** Omar Soliman El Safoury, Dina Sabry Abd El Fatah, Magdy Ibrahim

**Affiliations:** *From the Department of Dermatology, Research Biostatistics Unit, Management Team, EBM Unit, MEDC, Cairo University, Egypt.*; 1 From the Department of Biochemistry, Research Biostatistics Unit, Management Team, EBM Unit, MEDC, Cairo University, Egypt.; 2 From the Department of Obstetrics & Gynecology, Research Biostatistics Unit, Management Team, EBM Unit, MEDC, Cairo University, Egypt.

**Keywords:** *D-penicillamine*, *eyelid cosmetics*, *kohl*, *galena*, *lead toxicity*, *periocular hyperpigmentation*

## Abstract

**Background:**

Periocular hyperpigmentation is a condition in which skin of eyelids become darker in color than the normal surrounding skin. Lead and other heavy metals produce increased pigmentation because of deposition of metal particles in the dermis and increased epidermal melanin production.

**Aims:**

This study was conducted to evaluate the dual effect of chelation therapy in treating periocular hyperpigmentation and lead toxicity.

**Methods:**

The study population consisted of nine females complaining from dark coloration of their eyelids. The nine females were continuously using kohl as eyeliner. Lead levels in conjunctiva and serum before and after D-penicillamine (D-PCN) oral administration were estimated in relation to vertical, horizontal length, and degree of hyperpigmentation score.

**Results:**

Highly significant *P* values (0.000) were obtained as regard to the conjunctival lead levels, serum lead levels, horizontal length, and degree of darkness score before and after D-PCN therapy. A less significant *P* value (0.040) was recorded as regard to the vertical length.

**Conclusion:**

Regardless other causes, this study spots the light on a new concept for periocular hyperpigmentation from lead toxicity in adult females using kohl and suggests D-PCN in a low divided dose (750 mg/day) for its treatment.

## Introduction

The traditional eye cosmetic known as kohl is an eye powder used for adding a measure of mystery and allure to the eyes of women. Originally, kohl in Arabia made of antimony trisulfide and the ore stibnite was called “ethmid.” As this was scarce and expensive, it was slowly replaced over the years by galena that is lead sulfide with the same gray-black color and shiny appearance like stibnite.[1] In a study conducted in Cairo using X-ray powder diffraction and scanning microscopy on 18 kohl samples used in the market showed galena (lead sulfide) in 6 samples.[2] Abnormal pigmentation of the conjunctiva as well as lacrimal sac was noted in those patients using eyeliners.[3] The peri-orbital discoloration is composed of fine to coarse extracellular pigment often surrounded by chronic inflammatory infiltrate, mainly lymphocytes with few plasma cells and neutrophils. Abnormal pigmentation of the conjunctiva and lacrimal sac was noted in the form of punctate black spots in the fornicial and tarsal conjunctiva of upper and lower eyelids. Focal to diffuse black pigmentation of lacrimal sac was also observed. Lead and other heavy metals produce increased pigmentation because of deposition of metal particles in dermis and increased epidermal melanin production.[4] Lead causes “lead hue” which is a mixture of pallor and lividity.[4] So, it induces or aggravates the grade of hyperpigmentation. Pigments can reach the skin of lower eyelids after tattooing by a phenomenon known as fanning and the same can occur from lead of kohl.

### The aim of this clinical study

To evaluate the dual effect of D-penicillamine (D-PCN) as a chelating agent in treating the periorbital hyperpigmentation and lead toxicity induced by lead of kohl.

## Materials and Methods

This study is an extension of a previous study[5] on kohl and its relation to periocular hyperpigmentation. The previous study included 54 females, 44 females with periocular hyperpigmentation. Twenty-two of them were using kohl and 22 were not using kohl and 10 were used as control. The participants were subjected to computed tomography scanning (CT scan) for maxillary, frontal sinuses and ocular globes, complete blood picture, serum, and conjunctival lead estimation. In order to reverse the lead levels in the previous study, chelation was used for nine females from the group using kohl. The study was conducted after approval of the Ethics and Research Committee of the University. The participants were regularly using kohl as eyeliner (mean 7.8 ± 6.95 years). Their mean age was 24 ± 7.98. According to the degree of darkness, the following scores were given. Score (1) for light brown (no participants), Score (2) for dark brown (5 participants), and Score (3) for brown black (4 participants).The importance of grade 1 pigmentation lies in follow-up after therapy. History of exposure to environmental sources of lead pollution was documented. A low dose of D-PCN may reduce the rate of adverse effects without a significant reduction in the drug's efficacy.[6] Each participant was given 750 mg daily in a divided dose instead of 900-1,800 mg daily (10.5 mg/kg/day which is less than the reported reduced dose of 15 mg/kg/day).[6]

### Analysis of serum and conjunctival lead concentrations

Skin biopsies for lead detection in skin of eyelids were refused from both participants and Ethics and Research Committee for cosmetic reasons. So, a conjunctival swab sample was taken and directly moistened in phosphate buffer saline (participants were instructed to stop kohl for at least 1 week before swabs). Estimation of lead both in serum and conjunctival swab were done by using flame furnace absorption spectrophotometer method.[7] A cold vapor generation technique was used to determine lead level with hallow cathode lamp and slit interval of 0.7 nm and at a wavelength of 283.3 nm. The continuum background correction was applied in all estimations by using D2 lamp. The limit of detection was estimated as three times the standard deviation for a 20 run of blank. The final concentration of lead was estimated from a standard curve.

## Results

The mean level of creatinine was 0.8 ± 0.21 (0.7-1.5 mg/dl). The mean hemoglobin level was 11.69 ± 1.19 (12-14 g/dl). Urine and blood abnormalities or systemic symptoms were not recorded with this low divided dose. Blood pressure was normal. The mean conjunctival lead level decreased from 34.02 ± 7.86 μg/ml to 12.11 ± 3.66 μg/ml after therapy with a highly significant *P* value of 0.000. The mean serum lead decreased from 35.36 ± 4.51 μg/ml to 12.27 ± 3.26 μg/ml with a mean *P* value 0.000. The mean width of the periocular hyperpigmentation decreased after therapy from 3.83 cm ± 0.50 to 2.44 cm ± 0.58 with a highly significant *P* value of 0.000, while the mean vertical length decreased from 2 cm ± 0.43 to 1.5 cm ± 0.50 with a significant *P* value of 0.04. The mean degree of periocular hyperpigmentation score decreased from 2.44 ± 0.53 to 1.67 ± 0.50 with a highly significant *P* value of 0.000. The correlation of decreased conjunctival and serum lead levels due to D-PCN therapy was non-significant. N.B; after 2 weeks: The mean conjunctival lead level decreased from 32.02 ± 7.86 μg/ml to 24.44 ± 2.88 μg/ ml. While the serum leads level decreased from 35.36 ± 4.51 μg/ml to 22.94 ± 2.94 μg/ml. According to the US centers of disease control and prevention guides (2005), this is higher than 20 μg/ml which is the recommended threshold limit value for renal affection. So the therapy was extended from 2 weeks to 1 month.

## Discussion

**Figure 1:**
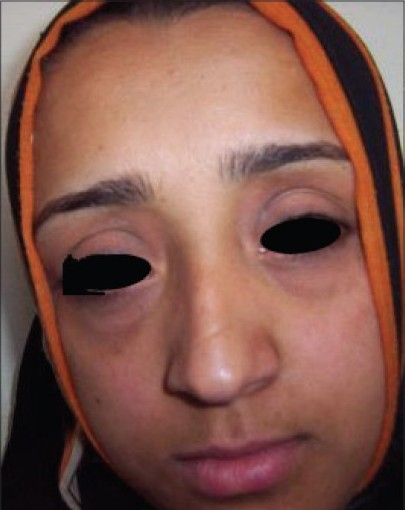
Before treatment

**Figure 2:**
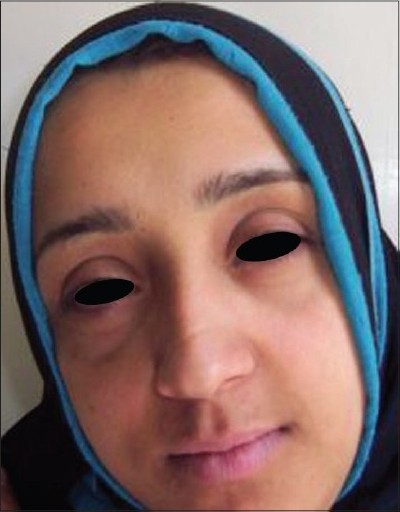
After treatment (Satisfactory)

**Figure 3:**
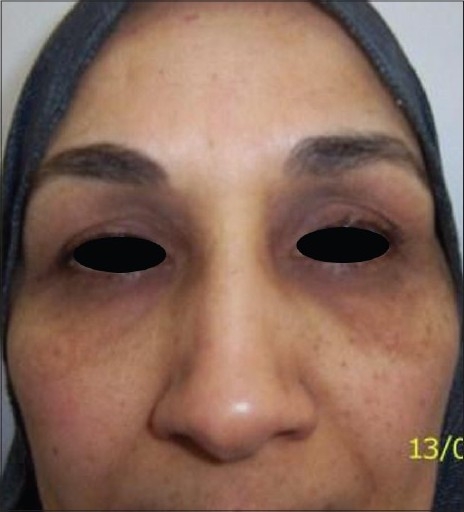
Before treatment

**Figure 4:**
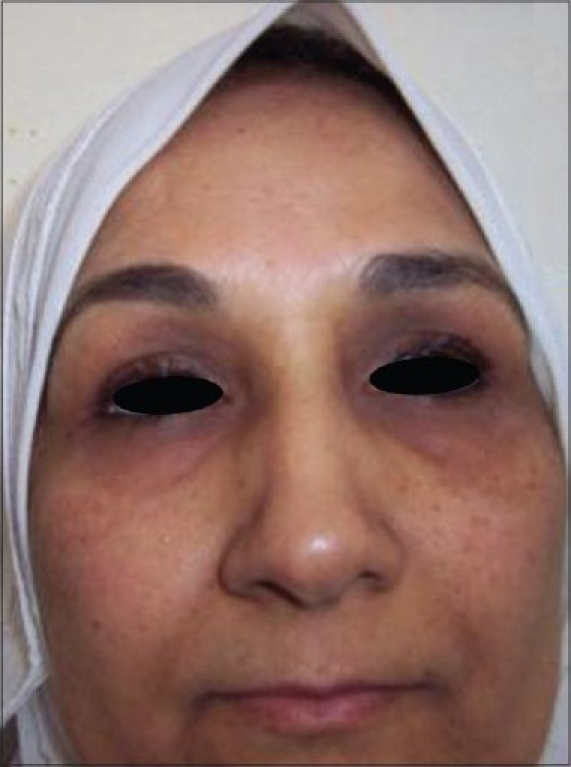
After treatment (Unsatisfactory)

The basis on which this clinical trial was conducted depended on a previous study of three groups of females with periocular hyperpigmentation, using kohl, not using kohl, and control. The mean values of lead obtained among the kohl users, non-kohl users and control were 31.16, 5.40, and 5.81 μg/ml in conjunctiva compared to 32.27, 17.23, and 18.01 μg/ml in serum. According to these results, the conjunctival lead was six times higher among females applying kohl, raising the serum lead of kohl users to double the serum lead levels among non-kohl users and controls. The positive correlation detected between conjunctival and serum lead was highly significant (*P* value 0.000), the more the lead level in conjunctiva, the more the serum level. Therefore, the results obtained suggest using kohl as a source of mild-to-moderate lead toxicity (highly significant *P* < 0.005-significant *P* < 0.05). In non-kohl users group, 9.09% showed grade (1) hyperpigmentation, 54.55% showed grade (2) hyperpigmentation, and 36.36% showed grade (3) hyperpigmentation. These data were comparable to 9.09%, 31.82%, and 59.09% among kohl-users, respectively. Comparing the degree of hyperpigmentation in both groups suggests that kohl is an aggravating factor for hyperpigmentation.[5] A highly significant correlation was obtained between frequencies of kohl application to the length of hyperpigmentation (*P* value 0.005) and to the degree of hyperpigmentation (*P* value 0.001). A positive statistical correlation was obtained between the duration of kohl application and width of hyperpigmentation (*P* value 0.021).[5] As lead toxicity is chronic, so to decrease lead levels, stopping kohl alone is illogic, hence chelation should be used. It was interesting that a significant reduction of the vertical and transverse dimensions of the eyelid hyperpigmentation occurred in concomitance with the decreased lead levels. Clinically, the hyperpigmentation faded in responders from lateral to medial side in width and from below to above in vertical length with a remnant of hyperpigmentation in the inner canthus. The decrease in pigmentation score was satisfactory to the participants in grade (2) hyperpigmentation [Figures 1 and 2], but was not satisfactory in grade (3) hyperpigmentation [Figures 3 and 4]. The follow-up of the participants for 1 year revealed no recurrence of hyperpigmentation.

Effective chelation therapy for intoxication by heavy metals depends on whether the chelating agents are able to reach the intracellular site where the heavy metal is firmly bound. The most widely used chelating agents are calcium disodium ethylenediamine tetra acetic acid (CaNa_2_EDTA), D-penicillamine, and British Anti-Lewisite (BAL). Meso 2, 3 dimercaptosuccinic acid (DMSA), an analogue of BAL, D-penicillamine had been used successfully in treatment of lead toxicity with excellent results. The non-significant correlation of serum and conjuctival lead indicates the ability of D-PCN to remove lead equally from serum and skin.

## Conclusion

This study spots the light on a new concept of periocular hyperpigmentation from lead toxicity in adult females using kohl for long time and suggests D-PCN in a low divided dose (750 mg/day) for its treatment.
